# Rejoinder to Commentaries on: On the Uses and Abuses of Regression Models: A Call for Reform of Statistical Practice and Teaching

**DOI:** 10.1002/sim.70065

**Published:** 2025-06-24

**Authors:** John B. Carlin, Margarita Moreno‐Betancur

**Affiliations:** ^1^ Clinical Epidemiology & Biostatistics Unit (CEBU) Murdoch Children's Research Institute Melbourne Victoria Australia; ^2^ CEBU, Department of Paediatrics The University of Melbourne Melbourne Victoria Australia; ^3^ Centre for Epidemiology & Biostatistics Melbourne School of Population & Global Health, University of Melbourne Melbourne Victoria Australia

We thank the discussants for their stimulating commentaries on our paper [1]. It was very gratifying to see their broad agreement with our observation that there are major problems with the way in which many analyses (almost universally based on regression models) are conceived, conducted and reported in the medical literature. Most agree with our central proposition that these problems can be traced to the way in which statisticians—and by extension, non‐statistician researchers—are taught, with a focus on models and methods ahead of sharp research questions. As Robert Platt (henceforth RP) [2] succinctly summarizes, “it is far too often that researchers start on a regression exercise without clear goals, leading to incoherent interpretation of regression coefficients […]”.

Despite broad agreement that there is a problem, some substantial differences in perspective are apparent. We address the major ones under the next three headings before touching on some additional points.

## 1 The Three Types of Question

It is not clear that all discussants agree with our fundamental starting point based on the “three tasks of data science” as articulated by Hernán et al. [3] Most of the discussants acknowledge the appeal of the “three tasks” framework, and RP, Shmueli (GS) [4] and Nold and Heinze (N&H) [5] broadly endorse its fundamental role. While we are sympathetic to many of Greenland's (SG) [6] wide‐ranging comments, we feel he began on a confusing note by referring to the ‘*classification of regressions* into “descriptive”, “predictive” and “causal”’. This would make sense if edited to the “classification of purposes for which regressions may be useful”. Sauerbrei et al. (S&S) [7] and GS fall into variations of the same trap, talking about a classification of “regressions” (e.g., “descriptive regressions”) rather than of research questions. In contrast, we emphasize the three‐tasks framework as an essential starting point, requiring classification of the primary research question as either descriptive, predictive or causal before using the roadmap approach (define estimand, delineate assumptions, THEN consider estimation), outlined in our paper and elsewhere [1, 8], to plan an analysis that may use regression methods in its final step.

S&S's contention that “the purpose of regression modeling tasks can be more complex than three types” seems to conflate the issue of the undeniable complexity that arises with many analysis tasks (such as in addressing causal questions, where regression models may be used in various ways, as they describe) with a suggestion (not fully elaborated) that the three‐tasks classification of question types (not regressions!) is not a helpful starting point. We are merely suggesting the latter—start from a clear question type and then address the complexity as needed, whether using regression as appropriate or other methods, as indeed outlined in the helpful STRATOS tutorial on causal inference (their ref. 12) [9]. In their argument that the three tasks are “not sufficient”, S&S seem to suggest two additional possible question types—“causal discovery,” which we address under the causality heading below, and “prognostic factor studies”.

To us, the latter provide an example of an area where the focus on statistical methods has usurped clarity of purpose in study aims. The aim of a prognostic factor study is said to be to identify factors that are “associated with” a subsequent endpoint (such as death), and such studies seek to do this by identifying variables that have an “independent association” with the outcome when included in a multivariable regression model. In a similar vein, GS's notion of a “descriptive model” (see her figure) suggests that in such a model, the regression coefficients for covariates that “remain in [the] model only if statistically significant” are of scientific interest (and thus a meaningful target of statistical inference). We strongly disagree with this, for two reasons. First and foremost, this classical interpretation conveys a strongly causal flavor, but that is fraught due to the “Table 2 fallacy” [10]. And if not a causal interpretation, then what? How are the results to be used meaningfully in the real world? A straightforward interpretation seems possible only if we believe that the process arrives at the true data generating model. But why should we believe that outcomes in humans are truly generated by multivariable linear regressions, let alone that statistical techniques can “discover” this model? Second, this approach implies, through the selection based on statistical significance, that substantive conclusions should depend (heavily!) on sample size.

Vansteelandt and Steen (V&S) [11] welcome our perspective that “the statistical model often overshadows the scientific inquiry itself” and agree that statistics education is in particular need of attention. However, they emphasize many challenges with the proposed framework, in particular suggesting that the distinction between the three tasks is not always clear‐cut. While that may be true in the initial consideration of a research task, ultimately assigning the research question to one of the three categories is both possible, by considering the intended use of findings (as highlighted by GS), and an essential initial starting point to then addressing it. With our historical Example 1, although the desired clarity was not present at the time of the initial work, it was clear that the investigators were focused on the question of characterizing differences in kidney size in order to better assess growth when interpreting future radiological scans—a descriptive intent. The question of interest would have been causal, as suggested by V&S, if the intent was instead to examine the potential impact on kidney size if infections were better controlled.

Allowing there to be a “continuum” of purposes, as V&S suggest, would in our view invite further confusion, because with no concrete starting point from which to plan an analysis, it is a slippery slope to “everything goes” and the purpose‐free application of methods that we witness now. Having said that, we agree it is important to acknowledge the existence of a fourth category (or rather a type of question that overlaps two categories), “prediction under intervention” [12], although we see this as essentially a causal inference task where method performance should focus on predictive ability instead of bias. Our Example 2 could have had this character, as suggested by V&S, but our understanding is that the usage of gas enema was not in question, only whether successful outcomes could be predicted. We return to discuss V&S's suggested distinction between “etiological” and “comparative effectiveness” analyses in the next section.

Recapping our key point, in our applied work and teaching we stick very closely to the three‐tasks framework, working with substance‐matter collaborators to reach clarity on whether their primary research question is descriptive, predictive or causal before tackling the question using the roadmap concept [1, 8]. Without this framework we believe confusion around concepts like “descriptive regression” and “prognostic factor studies” will persist.

Aside from causal questions (see Section 2), the other type that seems to remain somewhat unclear among discussants is the descriptive question, so we reiterate that a descriptive research question is one that seeks to characterize one or more features of the distribution of an attribute or health problem across the population (with the ultimate purpose to better understand a health burden or risk). Once the target feature(s) and population are clearly defined, then our roadmap approach can proceed [8].

## 2 Perspectives on Causality

Several discussants touch on philosophical issues underlying the study of causality. For example, GS makes a distinction between ‘“causal description” (what is the effect?) and “causal explanation” (why?)’. There are of course different views but, like others [13], we believe the latter task lies outside the scope of empirical studies for which statistical methods might be used. Therefore our focus is squarely on the former, which we understand to be the domain of “causal inference” as considered in our paper, relating to the study of “what if” questions, about the effects of interventions [14]. In this light, “causal description” seems a confusing term; if an alternative to “causal inference” is desired, then “causal quantification” seems preferable.

V&S propose a distinction between “etiological” and “comparative effectiveness” analyses based on the stage of the research area, specifically the availability of well‐defined interventions. Per the above, the latter is very close to what we understand and describe as causal inference, while the former is harder to understand, as it implies that causes can be “discovered” by statistical analysis, as implied by the example of the widely discussed article they cite [15]. As discussed in our paper and elsewhere [10], it is not logically possible for a single multivariable regression model to answer causal questions about a list of putative causal effects. Vansteelandt's proposed “assumption‐lean modeling framework” for tackling these problems while avoiding “Table 2 fallacy”‐type critiques is interesting but cannot in our view surmount the impossibility of discovering causes. In particular, we note that the assumption‐lean model they specify for studies of causal risk factors refers to potential outcomes under interventions that set these factors, so it seems to remain subject to issues with consistency violations in the absence of well‐defined interventions. Even at a relatively early stage of investigation when numerous putative “causes” may be of interest, we believe the formal causal inference framework as described in our paper is needed. This makes the findings of such research relevant to inform future causal inference research (e.g., to develop and/or evaluate actual interventions). That is, we believe that the fact that a research area is at an “exploratory” stage does not justify failing to address these questions in a way that is most appropriate to connect to the potential future use of the findings. It just means that the findings need to be interpreted cautiously, and in fact following a formal causal inference framework assists in making the limitations transparent, and this understanding can also be used to design better studies in the future.

The previous comments address S&S's concern that we failed to elaborate on what we meant by the distinction between settings “where little is known” and “where much is known”. S&S also mention the task of discovering the causal structure in high‐dimensional problems. However, we are skeptical of claims that it is possible to discover the “true DAG” from analysis of data, because of our disbelief that it is possible to discover causes (i.e., answer “why?” questions) using statistical analysis.

## 3 What Is a “Good Model” When We Let Go of the True Model Myth?

Our critique of current teaching and practice revolves largely around what we call the true model myth, the notion that the core task of the statistician is to build a model that closely approximates the true data‐generating process. Once we step away from this and take our preferred roadmap approach, then what is a “good model” depends directly on the type of question that we are seeking to answer. For example, in the estimation of causal effects, we should aim to develop models and methods that focus first and foremost on reducing potential bias, with a key example being the need to control for confounding in observational studies. In this light, generic concerns about “good” models as reflected in traditional model‐checking “diagnostics” may be a distraction, encouraging thinking that mirrors the true model myth rather than focusing on potential sources of bias with respect to the study aims.

For example, N&H propose that “the key is to teach how to develop a defendable model”, with “diagnostics” playing an important role in this. We emphasize that the first “diagnostic” should be whether there is clarity around how a proposed model might answer a sharply defined and relevant research question, after which study design and causal assumptions (including in the broad sense implied by SG's concept of the “causal generation of data”) must be considered. Ultimately, the statistician will consider models and may find technical tools such as the examination of residuals helpful in improving a model specification, but the important question is whether it leads to better predictions in the target population (for a prediction question) or unbiased and more precise estimation of a causal effect (causal question) or descriptive estimand (descriptive question) under the causal (and potentially parametric) assumptions deemed plausible, rather than whether the model itself is somehow closer to an ideal that textbooks suggest.

Consistently with our view, SG rightly emphasizes that models are never right or wrong and need to be evaluated in the context of the purpose at hand and external scientific knowledge as well as traditional statistical tools such as inspection of residuals. He also raises excellent points about the dangers of sparse data, a problem particularly underappreciated in causal inference and which we only briefly touched on in the paper. This certainly warrants broader attention in the future, potentially using developments in causal machine learning to circumvent parametric modeling constraints.

## 4 Additional Points

Before concluding, we touch on several other points raised in the commentaries. Among those that echo our themes, RP nicely highlights another problem with current biostatistical teaching: that the focus on models has arguably distracted from focusing on underlying purposes by aiding and abetting the continuing popularity of the odds ratio parameter, despite its well‐documented difficulties [16]. He also observes that the traditional focus on regression models reflects the continuing emphasis in our teaching and training on analysis over study design, which we agree is problematic, noting the prominent role of design issues such as measurement and sampling and related causal assumptions (in the broad sense of SG) within our roadmap concept.

GS's comments provide a nice bridge from our focus in biostatistics to her area of management and social science, and thereby implicitly to other areas of empirical investigation. Further interesting observations are made about the importance of considering the “unit of action” (the individual or the “collective”) and the important role of software, to which we would add that the continuing proliferation of software tools sharpens the challenges that statisticians face in providing analytic guidance, with increasing emphasis on the need to understand the software and to guard against the misuse that readily occurs in the absence of strong understanding of underlying principles and methods.

SG canvasses a wide range of related topics, in a largely complementary vein to our paper, while agreeing with our central thrust and sharing our concerns about the current state of teaching and practice. Although, as already discussed, we felt his concept of “descriptive regression” was confusing, we agree that the ideas he describes in the vein of regression models as “data reductions” are useful. However, they need to be handled carefully because of the ever‐lurking dangers of perpetuating the widespread practice of fitting models without a prespecified purpose and then back‐engineering substantive interpretations of the results. We agree with his concerns about the underrecognized gap between conventional statistical inferences directed at an assumed “data generator” and truly engaged analysis directed at underlying target population quantities, which echo RP's comments on the need for making study design and related assumptions more prominent in teaching and practice. We also appreciate his thoughts on some of the details that are often overlooked in the careful construction of models for various purposes, including the handling of continuous variables, although these points beg some questions around modeling that targets meaningful causal and plausibly identifiable estimands in the context of continuous exposures. His Closing Cautions highlight the fact that the issues we discuss are deep and difficult and deserve to be taken seriously by teachers of biostatistics at all levels, with teaching at the more technical levels undermined by what he terms a “math delusion” that leaves many biostatistics graduates poorly equipped to engage with messy applied problems.

V&S also provide a fascinating set of comments, to many of which we have already responded. A specific further concern is some disquiet with the approach that we took to our first example, where, as already mentioned, we in effect back‐engineered our roadmap approach to an analysis that had been done many years earlier within a less clearly defined framework. In the paper, we discussed at some length what the first author (with admittedly some reservations from the second author!) considered a defensible detour on the proposed roadmap for descriptive questions, which allows consideration of the potential for regression modeling adjustment to provide a more precise estimate of the target parameter compared to a simple estimate that remains closer to the raw data. This detour clearly raises some of the dangers of allowing questions to follow models that we warn against, and we agree with V&S's concerns about the need for a strong rationale for covariate adjustment in descriptive studies (having ourselves cited some of the same papers that they cite). In descriptive studies, it may often be best to focus on guarding against bias, even if that comes at the expense of reduced precision. With a larger sample of data in an example such as this, where the age covariate is so important, it might be possible simply to examine age‐specific differences. However, we hope the approach we described provides some insight and intuition into the connections between model‐free and model‐based estimation (see further detail in Supporting Information of our paper [1]).

We were somewhat mystified by N&H's title, because our paper did not intend to focus on “Teaching Statistics as a Minor Subject”. Our proposal is that the teaching of statistics to biostatistics “majors”, whether at advanced undergraduate or Masters level, needs to be radically reformed, with the hope that these reforms would lead to “trickle‐down” benefits for “minor” students and others. However, we do agree that greater emphasis on the role of statistics within science, as discussed in N&H's final paragraph, is important at all levels, including specialist training.

It was disappointing that S&S felt that we were concerned with an insufficiently broad scope of “epidemiological methodology”, while we intended our concerns to encompass biostatistical work in considerable generality, across the full range of epidemiological investigations, conceived broadly to include “clinical epidemiology” (and thus clinical trials), for instance. Similarly to N&H, these authors highlight the distinction between “(bio)statisticians and users of statistical methods” and imply that the main problems lie with the latter, but this seems to be shirking our responsibility as statisticians. We appreciate of course that these authors have worked hard over many years to improve statistical standards, especially via the STRATOS initiative. However, it can surely not be true that all guidelines are equally helpful. In particular, we would say that the STRATOS guidelines directed at analyses pursuing clearly defined purposes, such as causal inference [9], are more useful than those directed at the ill‐defined purpose (discussed above) of “building descriptive regression models” [17]. Another example of guidelines that don't necessarily improve practice is provided by the PROGRESS initiative [18], which aimed to provide guidance on “prognostic factor studies”, which we do not consider to have a meaningful research purpose, as discussed above. Perhaps a new guideline is needed (a guideline to rule the guidelines?!) on how to approach analysis planning using the three types of research question—this could build on an analysis planning template that we have published [19], alongside detailed roadmaps for each type of question and examples of usage [8].

Finally, S&S raise the interesting topic of “initial data analysis (IDA)”, suggesting that this needs to be included in the roadmap for design of analyses. We agree that it is important to formalize a process for enabling deviations from analysis plans when data issues arise, and a systematic approach to data checking and verification would be valuable. However, we believe this should be incorporated into the three‐tasks framework, and indeed the authors' recent guide to this approach (their ref. 7) appears to be situated within the framework of prediction modeling.

## 5 Conclusion

In closing, we once again challenge the discussants and readers to consider the examples listed in our brief review of three medical research journals, especially the prevalence of analyses pursuing ill‐defined questions. We can lament this and agree that it is partly due to inadequate understanding on the part of the legions of “amateur” statisticians and data analysts who perform analyses for many medical studies. But, as mentioned above, our reform proposal is not primarily aimed at non‐specialist statisticians: we are convinced that the problems stem from our own profession's insufficient engagement with the underlying issues. Too many of our graduates, even at Masters and PhD level, approach their early collaborative experiences with the promise that they can “fit a good model”, rather than first asking the collaborator “now, what exactly is the question you are trying to answer? Let's talk about that…”.

How can we remedy these problems? We are convinced that this requires substantial, if not wholesale, changes to the curricula of biostatistical training programs. This will not be easy to achieve, but our efforts to date suggest that it may be approached in stages, beginning with introductory courses and progressing to reorganize the more technically oriented courses to which biostatistics students' progress. Noting the textbook challenge raised by GS, we are very much aware that this reform would be greatly facilitated by the development of relevant teaching materials.

## Conflicts of Interest

The authors declare no conflicts of interest.

## Data Availability

Data sharing not applicable to this article as no datasets were generated or analysed during the current study.

## References

[1] J. B. Carlin and M. Moreno‐Betancur , “On the Uses and Abuses of Regression Models: A Call for Reform of Statistical Practice and Teaching,” Statistics in Medicine 44, no. 13‐14 (2025): 1–16, 10.1002/sim.10244. PMC12186762 40553044

[2] R. Platt , “Regression—A Means, Not an End,” Statistics in Medicine 44, no. 5 (2025): 1–2, 10.1002/sim.70000. PMC11866495 39905855

[3] M. A. Hernán , J. Hsu , and B. Healy , “A Second Chance to Get Causal Inference Right: A Classification of Data Science Tasks,” Chance 32, no. 1 (2019): 42–49.

[4] G. Shmueli , “To Explain, to Predict, or to Describe: Figuring out the Study Goal,” Statistics in Medicine 44, no. 13‐14 (2025): 1–4, 10.1002/sim.10307. 40553038

[5] M. Nold and G. Heinze , “Commentary: Teaching Statistics as Minor Subject—Handing on Fire, Not Worship of Ashes,” Statistics in Medicine 44, no. 13‐14 (2025): 1–2, 10.1002/sim.10284. PMC12186761 40553048

[6] S. Greenland , “Some Ways to Make Regression Modeling More Helpful than Misleading,” Statistics in Medicine 44, no. 13‐14 (2025): 1–6, 10.1002/sim.10313. 40553052

[7] W. Sauerbrei , F. Ambrogi , R. de Bin , A.‐L. Boulesteix , E. Goetghebeur , and M. Huebner , “Regression Models—Efforts Are Required to Improve Statistical Practice and Teaching,” Statistics in Medicine 44, no. 13‐14 (2025): 1–3, 10.1002/sim.10341. PMC12186759 40553039

[8] D. A. Shepherd , D. J. Amor , and M. Moreno‐Betancur , “Statistical Analysis of Observational Studies in Disability Research,” Developmental Medicine and Child Neurology 66 (2024): 1408–1418. 38721699 10.1111/dmcn.15948

[9] E. Goetghebeur , S. le Cessie , B. De Stavola , E. E. Moodie , I. Waernbaum , and the topic group Causal Inference (TG7) of the STRATOS initiative , “Formulating Causal Questions and Principled Statistical Answers,” Statistics in Medicine 39, no. 30 (2020): 4922–4948. 32964526 10.1002/sim.8741 PMC7756489

[10] D. Westreich and S. Greenland , “The Table 2 Fallacy: Presenting and Interpreting Confounder and Modifier Coefficients,” American Journal of Epidemiology 177, no. 4 (2013): 292–298. 23371353 10.1093/aje/kws412 PMC3626058

[11] S. Vansteelandt and J. Steen , “Discussion of ‘On the Uses and Abuses of Regression Models: A Call for Reform of Statistical Practice and Teaching’,” Statistics in Medicine 44, no. 13‐14 (2025): 1–5. 10.1002/sim.10312 40553079

[12] R. H. Keogh and N. Van Geloven , “Prediction Under Interventions: Evaluation of Counterfactual Performance Using Longitudinal Observational Data,” Epidemiology 35, no. 3 (2024): 329–339. 38630508 10.1097/EDE.0000000000001713 PMC11332371

[13] M. A. Hernán , “Does Water Kill? A Call for Less Casual Causal Inferences,” Annals of Epidemiology 26, no. 10 (2016): 674–680. 27641316 10.1016/j.annepidem.2016.08.016 PMC5207342

[14] M. A. Hernán and J. M. Robins , Causal Inference: What if (Chapman & Hall/CRC, 2020).

[15] E. J. Williamson , A. J. Walker , K. Bhaskaran , et al., “Factors Associated With COVID‐19‐Related Death Using OpenSAFELY,” Nature 584, no. 7821 (2020): 430–436. 32640463 10.1038/s41586-020-2521-4 PMC7611074

[16] S. Greenland , J. M. Robins , and J. Pearl , “Confounding and Collapsibility in Causal Inference,” Statistical Science 14, no. 1 (1999): 29–46.

[17] W. Sauerbrei , A. Perperoglou , M. Schmid , et al., “State of the Art in Selection of Variables and Functional Forms in Multivariable Analysis—Outstanding Issues,” Diagnostic and Prognostic Research 4, no. 1 (2020): 3, 10.1186/s41512-020-00074-3. 32266321 PMC7114804

[18] R. D. Riley , J. A. Hayden , E. W. Steyerberg , et al., “Prognosis Research Strategy (PROGRESS) 2: Prognostic Factor Research,” PLoS Medicine 10, no. 2 (2013): e1001380. 23393429 10.1371/journal.pmed.1001380 PMC3564757

[19] M. Downes , D. Shepherd , R. Wijesuriya , et al., Statistical Analysis Plan Template for Observational Studies (2023), https://figshare.unimelb.edu.au/articles/online_resource/Analysis_plan_template_for_life‐course_cohort_studies/12471380.

